# One-year postoperative skeletal stability of 3D planned bimaxillary osteotomies: maxilla-first versus mandible-first surgery

**DOI:** 10.1038/s41598-019-39250-x

**Published:** 2019-02-28

**Authors:** Jeroen Liebregts, Frank Baan, Pieter van Lierop, Martien de Koning, Stefaan Bergé, Thomas Maal, Tong Xi

**Affiliations:** 1 Department of Oral and Maxillofacial Surgery, Radboud University Nijmegen Medical Centre, Geert Grooteplein 10, 6525 GA Nijmegen, The Netherlands; 2 Department of Orthodontics and Craniofacial Biology, Radboud University Nijmegen Medical Centre, Philips van Leydenlaan 25, 6525 EX Nijmegen, The Netherlands; 3 Radboudumc 3D Lab, Radboud University Medical Center, Nijmegen, The Netherlands

## Abstract

Orthognathic surgery is carried out to correct jaw deformities and to improve facial aesthetics. However, controversy surrounds whether the maxilla- or mandible-first surgery approach leads to better surgical outcomes. In our previous study, we have shown that in most instances, the maxilla-first surgical approach yielded closer concordance with the 3D virtual treatment plan than a mandibular-first procedure. However, the post-operative stability of each approach has not been investigated. Therefore, this one-year follow-up study was set-up and investigated the postoperative skeletal stability of the 3D planned translations and rotations after either the maxilla- or mandible-first surgery. In total, 106 patients who underwent bimaxillary surgery and had an individualized 3D virtual operation plans, received either maxilla-first (n = 53) or mandible-first (n = 53) surgery. 3D printed interocclusal splints were used during surgery to position the jaws. One year postoperatively a cone-beam computed tomography (CBCT) scan was made to assess the effects using the OrthoGnathicAnalyser. The mean sagittal, vertical and transverse relapse was less than 1.8 mm and no significant differences were found in relapse between the maxilla-first or the mandibular-first surgical procedure. Overall, this study shows that 3D virtual planning in combination with an optimised sequencing of osteotomies provides predictable long-term results in bimaxillary surgery.

## Introduction

In the past decade, significant controversy has surrounded the surgical approach taken during orthognathic surgery (corrective jaw surgery), in particular the sequencing of bimaxillary osteotomies. Traditionally, surgeons have opted to first operate on the maxilla, and secondly correct the osteotomies in the mandible. However, more recently several publications have highlighted the benefits of adopting the mandible first sequencing protocol, particularly in the downgrafting of the maxilla and a counterclockwise (CCW) rotation of the jaws^1–5^. Yet, there is little consensus on whether the maxilla-first or mandible-first surgical approach is more advantageous in terms of predictability and long-term stability of the postoperative results.

To obtain a harmonious facial profile and a stable dental occlusion, there has been an increase in using computer-assisted virtual surgical planning software in order to improve the predictability of the postoperative outcomes in orthognathic surgery^6^. An accurate transfer of the 3D planned jaw positions to the patient is required to achieve the virtually planned positions of the jaws at the end of the operation. Recently our group has demonstrated that using the maxilla-first surgical approach, the 3D planned translational and rotational movements of the maxilla and mandible can be accomplished more accurately, compared to the mandibular-first approach^4^. However, in cases of bimaxillary CCW pitch, the mandible-first surgical approach is preferred because this sequence results in more predictable displacements of the jaws^3,4^.

The postoperative skeletal stability is a major concern in obtaining satisfactory long-term results following bimaxillary osteotomies. Skeletal relapse is frequently reported, with an incidence varying between 2.0% and 50.3%^7^, and as a result the maxilla and mandible tend to return to their preoperative positions, leading to an enlarged overbite, malocclusion and deteriorating facial aesthetics. Relapse is associated with surgery related factors, such as the magnitude of the surgical displacement of jaws and the used surgical technique. However, there is no published evidence on the association between surgical approaches (maxilla-first or mandible-first) on the postoperative skeletal stability. Therefore, this study has evaluated the one-year postoperative skeletal stability of 3D planned bimaxillary osteotomies in patients who underwent either maxilla- or mandible-first surgical protocols.

## Results

The clinical cohort consisted of patients who underwent bimaxillary osteotomies at Radboud University Medical Centre between 2010 and 2014 (n = 116)^4^. In this one-year follow-up study, data from 106 patients (n = 73 female (69%); n = 33 male (31%); mean age 28 (range 16–57; Table 1)) were analysed to determine the level of skeletal relapse after undergoing either maxilla-first (n = 53) or mandible-first (n = 53) bi-maxillary surgery. In 57 patients an additional genioplasty was performed (maxilla-first n = 30; mandible-first n = 27). The patient cohort included 28 patients (26%), which had undergone a previous surgically assisted rapid maxillary expansion (SARME) prior to their bimaxillary surgery (maxilla-first n = 12; mandible-first group n = 16; Table 1). The post-operative CBCT-scan was acquired at 10.2 ± 3.0 months following surgery.

**Table 1:** Age, gender and surgical difference distribution within the study population.

	Maxilla-first surgery	Mandible-first surgery
Population (n = 106)	53 (50%)	53 (50%)
Age		
Mean	28.3	28.3
SD	11.3	10.9
Range	16–57	16–55
Male (n = 33)	14 (26%)	19 (36%)
Female (n = 73)	39 (74%)	34 (64%)
SARME in history	12 (23%)	16 (30%)
Genioplasty	30 (57%)	27 (51%)

SD: Standard Deviation.

### Overall skeletal relapse

The overall postoperative skeletal relapse of the maxilla and mandible in terms of translation and rotation are shown in Table 2. In patients who underwent the maxilla-first surgical approach, only the cranial/caudal translational movements showed a significant post-operative relapse (cranial: 0.7 ± 1.1 mm, p < 0.01; caudal: 0.7 ± 1.4 mm, p < 0.01). The remaining translational movements in the maxilla (left/right, anterior/posterior), were <0.3 mm and did not reach statistical significance.

**Table 2:** Translations and rotations of the maxilla and mandible after 1 week, 1 year and the postoperative relapse. Translations are given in millimetres, rotations are given in degrees.

		Maxilla					Mandible				
		n	CBCT 1 wk Mean ± SD	CBCT 1 yr Mean ± SD	Relapse (1wk-1yr) Mean ± SD	p-value	n	CBCT 1 wk Mean ± SD	CBCT 1 yr Mean ± SD	Relapse (1wk-1yr) Mean ± SD	p-value
**Translation**											
X	Left	51	1.4 ± 1.1	1.3 ± 1.2	0.20	0.20	62	1.6 ± 1.5	1.1 ± 1.7	0.5 ± 1.3	**0.00**
	Right	55	1.2 ± 1.2	1.1 ± 1.2	0.11	0.11	44	1.9 ± 1.8	1.3 ± 2.0	0.7 ± 2.0	0.04
Y	Anterior	97	3.3 ± 2.1	3.1 ± 2.1	0.19	0.19	92	8.1 ± 3.8	7.6 ± 3.2	0.5 ± 2.3	0.05
	Posterior	9	0.7 ± 0.5	0.5 ± 1.2	0.56	0.56	14	3.1 ± 1.4	1.3 ± 1.9	1.8 ± 1.2	**0.00**
Z	Caudal	57	2.8 ± 2.0	2.1 ± 1.9	**0.00**	**0.00**	52	3.2 ± 1.9	1.8 ± 2.3	1.4 ± 2.0	**0.00**
	Cranial	48	3.0 ± 2.3	2.2 ± 2.3	**0.00**	**0.00**	54	3.0 ± 2.4	2.2 ± 2.8	0.8 ± 2.0	**0.00**
**Rotation**											
Pitch	CCW	58	3.0 ± 2.7	2.0 ± 2.7	**0.00**	**0.00**	70	3.9 ± 3.1	1.7 ± 2.9	2.3 ± 2.6	**0.00**
	CW	48	3.5 ± 2.5	2.6 ± 2.5	**0.00**	**0.00**	36	4.0 ± 3.1	3.2 ± 3.1	0.8 ± 1.9	**0.02**
Roll	CCW	52	1.6 ± 1.5	1.1 ± 1.5	**0.00**	**0.00**	46	1.5 ± 1.5	0.9 ± 1.4	0.6 ± 1.1	**0.00**
	CW	54	1.1 ± 0.8	0.8 ± 1.0	**0.01**	**0.01**	59	1.2 ± 1.1	0.5 ± 0.9	0.8 ± 1.1	**0.00**
Yaw	CCW	52	1.3 ± 1.0	1.0 ± 1.1	**0.00**	**0.00**	57	1.3 ± 1.0	1.1 ± 1.4	0.2 ± 1.3	0.20
	CW	53	1.1 ± 1.1	0.9 ± 1.2	**0.03**	**0.03**	49	1.6 ± 1.5	1.3 ± 1.6	0.4 ± 1.1	**0.03**

CBCT: Cone-Beam Computed Tomography, SD: Standard Deviation, 1wk: one week, 1 yr: one year, CW: Clockwise, CCW: Counterclockwise.

For the overall translational directions of the mandible, only the posterior (1.8 ± 1.2 mm, p < 0.01) and caudal (1.4 ± 2.0 mm, p < 0.01) translations displayed relapses greater than 1 mm. For the rotational movements of the maxilla the postoperative relapse was below 1°, except for the pitch which showed the largest skeletal relapse (CW (=clockwise) 1.0° ± 1.3°, p < 0.01; CCW (=counterclockwise) 0.9° ± 1.6°, p < 0.01). The same trend was seen in the mandible, where the pitch was associated with the largest skeletal relapse (CW 0.8° ± 1.9°, p = 0.02; CCW 2.3° ± 2.6°, p < 0.01).

### Relapse maxilla-first approach versus mandible-first approach

**Figure 1:**
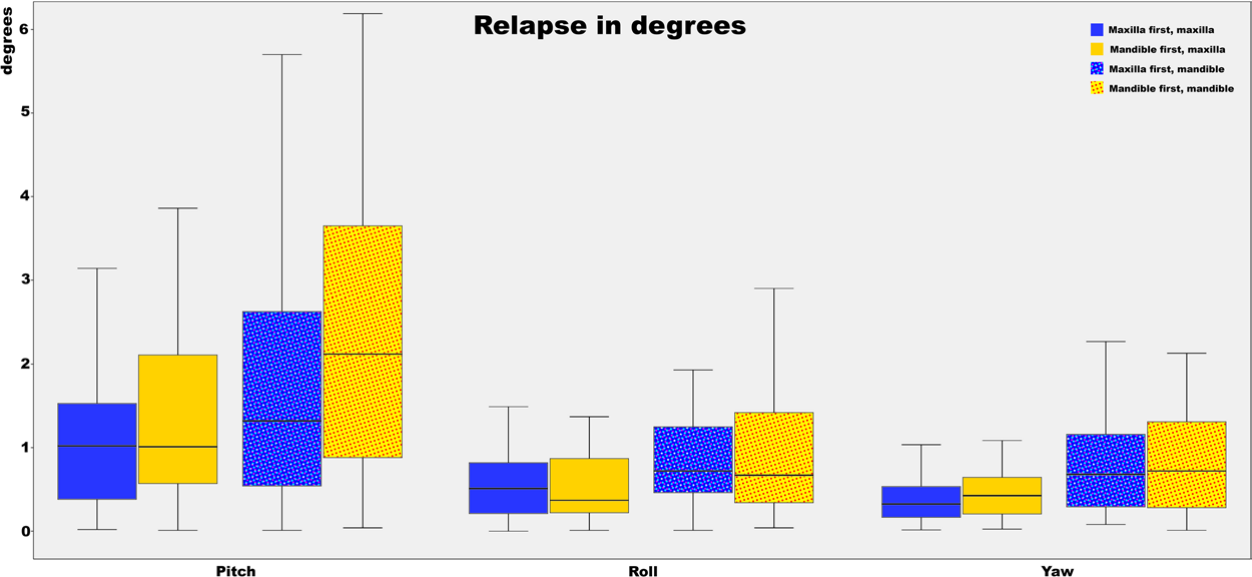
Boxplot of the differences between planned rotations and the postoperative outcome for the mandible and maxilla. Both the maxilla- and mandible-first groups are displayed in the boxplot. The whiskers of the boxplot represent the 25^th^ and 75^th^ percentiles. For the pitch the largest deviation is seen, the smallest deviation is seen in the roll. A negative pitch means that the achieved pitch is larger than the planned pitch. The same applies for the roll and yaw.

**Figure 2:**
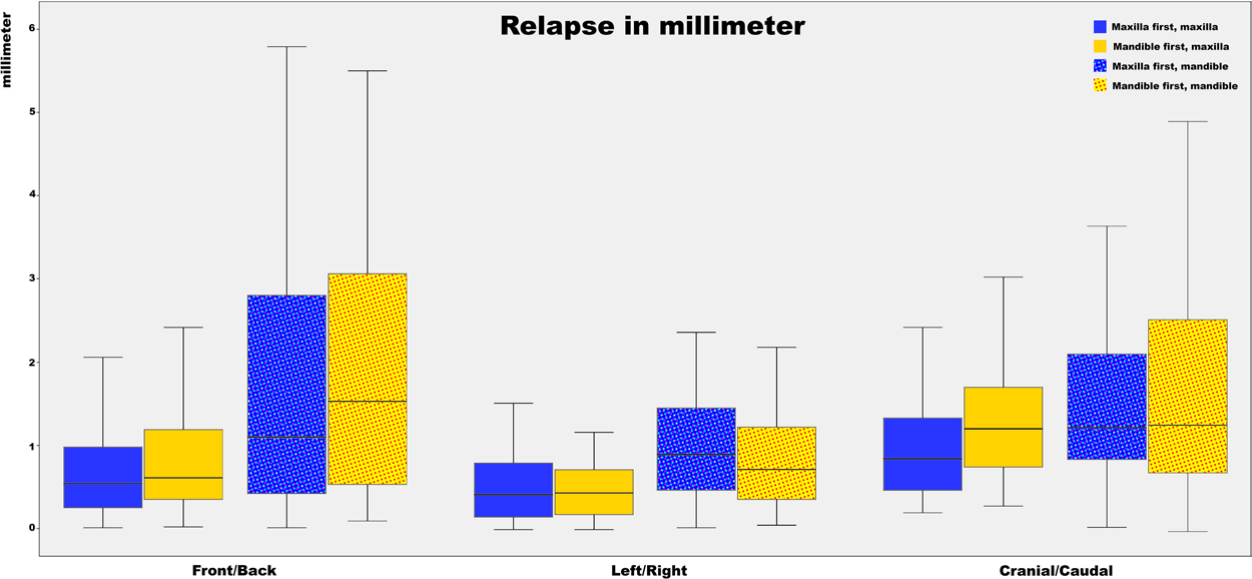
Boxplot of the differences between planned translations and the postoperative outcome for the mandible and maxilla. Both the maxilla- and mandible-first groups are displayed in the boxplot. The whiskers of the boxplot represent the 25^th^ and 75^th^ percentiles. The largest deviation is shown in the front/back translation, the smallest deviation is shown in the left/right translation. A negative front/back translation means that the achieved front/back translation is larger than the planned front/back translation; the same goes for the left/right and cranial/caudal translation.

The postoperative skeletal relapse of the maxilla and mandible for both the maxilla-first and mandible-first group, in terms of pitch, roll and yaw, and in terms of sagittal, vertical and transverse translations are shown in Figures 1 and 2. With regard to rotational movements, the pitch showed the largest rotational relapse in both the maxilla-first (CW 0.6° ± 1.4°, p = 0.04; CCW 0.6° ± 1.2°, p = 0.02) and mandible-first group (CW 1.4° ± 1.8°, p < 0.01; CCW 1.2° ± 1.2°, p < 0.01). For the translational directions of the maxilla, the median relapses of all directions are less than 1 mm except for the cranial/caudal displacement in the mandible-first group (median = 1.4 mm). As for the mandible, the largest relapse is seen in the front/back direction in both the maxilla-first (median = 1.1 mm) and mandible-first (median = 1.5 mm) groups.

No significant differences were found for the skeletal relapse of the maxilla between the maxilla-first and mandible-first groups (Table 3). As for the mandible, the maxilla-first group displayed significant less relapse concerning the CCW pitch compared to the mandible-first group, 1.6 ± 2.6° and 2.9 ± 2.5° (p = 0.04), respectively (Table 4). A statistical significant difference was also found in the mandibular relapse of the posterior displacement (p = 0.02), in favour of the maxilla-first group.

**Table 3:** Surgical displacements directly after surgery, one year after surgery and the relapse between one week and one year after surgery in the maxilla for both the maxilla-first group and the mandible first group. Translations are given in millimeter, rotations are given in degrees.

		Maxilla first					Mandible first					
		n	CBCT 1 wk Mean ± SD	CBCT 1 yr Mean ± SD	Relapse Mean ± SD	p-value*	n	CBCT 1 wk Mean ± SD	CBCT 1 yr Mean ± SD	Relapse Mean ± SD	p-value*	p-value**
**Translation**												
X	Left	29	1.7 ± 1.1	1.5 ± 1.3	0.2 ± 1.0	0.36	22	1.2 ± 0.9	1.01 ± 1.0	0.2 ± 0.8	0.36	0.94
	Right	24	1.3 ± 1.3	1.1 ± 1.3	0.3 ± 0.9	0.13	31	1.2 ± 1.1	1.07 ± 1.2	0.1 ± 0.7	0.49	0.37
Y	Anterior	49	3.8 ± 2.2	3.6 ± 2.2	0.1 ± 1.3	0.45	48	2.8 ± 1.8	2.55 ± 1.8	0.2 ± 1.3	0.28	0.80
	Posterior	4	0.4 ± 0.3	0.2 ± 1.8	0.2 ± 1.7	0.85	5	0.9 ± 0.6	0.66 ± 0.4	0.3 ± 0.7	0.40	0.91
Z	Caudal	29	3.1 ± 2.0	2.6 ± 1.8	0.5 ± 1.1	**0.02**	29	2.4 ± 2.0	1.45 ± 2.0	1.0 ± 1.6	**0.00**	0.17
	Cranial	24	3.3 ± 2.4	2.8 ± 2.7	0.5 ± 1.2	0.05	24	2.6 ± 2.1	1.66 ± 1.8	0.9 ± 0.9	**0.00**	0.19
**Rotation**												
Pitch	CCW	25	2.5 ± 2.6	1.9 ± 3.2	0.6 ± 1.2	**0.02**	33	3.2 ± 2.9	2.05 ± 2.3	1.2 ± 1.2	**0.00**	0.09
	CW	28	3.6 ± 2.7	3.0 ± 2.8	0.6 ± 1.4	**0.04**	20	3.4 ± 2.1	1.94 ± 1.9	1.4 ± 1.8	**0.00**	0.08
Roll	CCW	29	1.7 ± 1.8	1.3 ± 1.8	0.4 ± 0.6	**0.00**	23	1.3 ± 1.1	0.92 ± 1.0	0.4 ± 0.8	**0.02**	0.83
	CW	24	1.2 ± 0.9	0.9 ± 1.1	0.3 ± 1.0	0.14	30	1.1 ± 0.8	0.81 ± 0.9	0.3 ± 0.6	**0.01**	1.00
Yaw	CCW	24	1.3 ± 1.1	1.0 ± 1.1	0.3 ± 0.4	**0.00**	29	1.2 ± 1.0	0.94 ± 1.1	0.3 ± 0.7	0.06	0.97
	CW	29	1.1 ± 1.0	0.9 ± 1.4	0.2 ± 0.9	0.23	24	1.2 ± 1.2	0.96 ± 1.1	0.3 ± 0.6	**0.03**	0.76

SD: Standard deviation, CBCT: Cone-Beam Computed Tomography, 1wk: one week, 1 yr: one year, CW: Clockwise, CCW: Counterclockwise. *p-value between surgical displacements 1 week after surgery and 1 year after surgery, **p-value of the differences in relapse between the mandible-first and maxilla-first group.

**Table 4:** Surgical displacements directly after surgery, one year after surgery and the relapse between one week and one year after surgery in the mandible for both the maxilla-first group and the mandible first group. Translations are given in millimeter, rotations are given in degrees.

		Maxilla first					Mandible first					
		n	CBCT 1 wk Mean ± SD	CBCT 1 yr Mean ± SD	Relapse Mean ± SD	p-value*	n	CBCT 1 wk Mean ± SD	CBCT 1 yr Mean ± SD	Relapse Mean ± SD	p-value*	p-value**
**Translation**												
X	Left	35	1.7 ± 1.5	0.9 ± 1.8	0.7 ± 1.5	**0.01**	27	1.5 ± 1.5	1.2 ± 1.6	0.3 ± 1.0	0.14	0.19
	Right	18	2.1 ± 2.4	1.4 ± 2.3	0.8 ± 2.5	0.22	26	1.8 ± 1.3	1.2 ± 1.8	0.6 ± 1.6	0.08	0.79
Y	Anterior	46	9.8 ± 3.9	9.0 ± 3.3	0.8 ± 2.3	**0.03**	46	6.5 ± 3.0	6.3 ± 2.5	0.2 ± 2.3	0.59	0.21
	Posterior	7	3.6 ± 1.7	2.5 ± 2.0	1.1 ± 1.0	**0.03**	7	2.6 ± 0.8	0.1 ± 0.8	2.5 ± 1.0	**0.00**	**0.02**
Z	Caudal	27	2.6 ± 1.4	1.4 ± 2.0	1.1 ± 1.6	**0.00**	25	3.8 ± 2.2	2.2 ± 2.6	1.6 ± 2.3	**0.00**	0.42
	Cranial	26	3.4 ± 2.7	2.8 ± 3.1	0.6 ± 2.0	0.14	28	2.6 ± 2.1	1.5 ± 2.4	1.1 ± 1.9	**0.01**	0.41
**Rotation**												
Pitch	CCW	36	3.5 ± 3.4	1.9 ± 3.5	1.6 ± 2.6	**0.00**	34	4.3 ± 2.6	1.4 ± 2.1	2.9 ± 2.5	**0.00**	**0.04**
	CW	17	2.7 ± 2.3	2.5 ± 2.4	0.3 ± 1.7	0.55	19	5.1 ± 3.4	3.8 ± 3.6	1.3 ± 2.1	**0.02**	0.12
Roll	CCW	26	1.7 ± 1.8	1.0 ± 1.5	0.7 ± 1.1	**0.00**	20	1.4 ± 1.0	0.9 ± 1.3	0.5 ± 1.1	0.05	0.65
	CW	27	1.3 ± 1.0	0.6 ± 0.9	0.7 ± 0.9	**0.00**	33	1.1 ± 1.1	0.3 ± 0.8	0.8 ± 1.2	**0.00**	0.91
Yaw	CCW	26	1.4 ± 1.1	1.1 ± 1.8	0.3 ± 1.5	0.29	31	1.2 ± 1.0	1.1 ± 1.1	0.2 ± 1.2	0.49	0.64
	CW	27	1.9 ± 1.6	1.4 ± 1.8	0.4 ± 1.2	0.07	22	1.3 ± 1.2	1.1 ± 1.3	0.3 ± 1.0	0.24	0.55

SD: Standard deviation, CW: Clockwise, CCW: Counterclockwise. *p-value between surgical displacements 1 week after surgery and 1 year after surgery, **p-value between the difference in relapse of the mandible-first and maxilla-first group.

### Prognostic factors for skeletal relapse

Univariate regression analysis was applied to explore the influence of different patient and surgery characteristics on skeletal relapse. The sequence of the surgery did not have an influence on skeletal relapse in both the maxilla and mandible. Among factors such as gender, age, magnitude of surgical advancement and the counterclockwise pitch movement of the maxilla and mandible, the magnitude of intraoperative displacement exhibited the highest explained variance (5.3–30.3%) for nearly all directions in both the maxilla and mandible. This indicated a larger amount of surgical jaw displacement which results in more postoperative relapse (B 0.160–0.451). The counterclockwise pitch of the maxilla, and in particular of the mandible, also had a relatively large influence on skeletal relapse, with an explained variance of 4.8% and 28.4% respectively.

## Discussion

Bimaxillary surgery is used to correct misaligned jawbones (osteotomies), resulting in both a balanced and a stable dental occlusion as well as a harmonious facial profile. Bimaxillary surgery with either early, or late onset postoperative instability (relapse) has been shown to obtaining satisfactory long-term results. This unintended surgical outcome may lead to postoperative changes both in terms of function and aesthetics and may significantly affect the patient’s overall quality of life. Early postoperative skeletal relapse occurs shortly (<6 months) after the initial surgery, due to suboptimal condylar seating or slippage at the osteotomie sites^7–10^. Late relapse, on the other hand, tends to occur from six to twelve months after surgery. The pathophysiology of delayed skeletal relapse differs from the acute setting, and is believed to occur due to certain patient characteristics, such as type and magnitude of the surgical displacements and condylar resorption^11–13^. Previous studies have shown that postoperative skeletal relapse may correlate with the patient’s anatomical characteristics^9,14–16^, the magnitude of surgical displacements^7,17^ the direction of jaw displacements^12^, the use of osteosynthesis materials^18,19^ and the role of condylar resorption^20,21^. However, it remains unclear if the sequencing of bimaxillary osteotomies (maxilla- or mandible-first) may also influence postoperative skeletal relapse. To the author’s knowledge, the current study is the first comparative work to address this topic.

The results of the present study demonstrated that after one year the sequence of osteotomies in bimaxillary surgery does not appear to influence the one-year postoperative skeletal relapse. The skeletal relapse in the maxilla-first and mandible-first groups was comparable, ranging between 0.1–1.0 mm for the maxilla and 0.2–1.6 mm for the mandible. Subgroup analyses showed that the only differences in skeletal relapse between the two groups were present in the CCW pitch and posterior movement of the mandible in favour of the maxilla-first group. As the mean difference in relapse between both groups for CCW pitch and posterior displacement of the mandible were 1.3° and 1.4 mm respectively, well below the clinically relevant threshold of 2° and 2 mm, it is unlikely that the sequence of surgery has a clinically significant impact on the long-term postoperative skeletal stability. The overall postoperative skeletal stability of the maxilla was greater than that of the mandible. This finding was consistent with previous studies^22–25^. Compared to the maxilla, the skeletal relapse of the mandible is additionally influenced by adaptive changes in the temporomandibular joints and condyles and is thus generally larger. In addition, the larger skeletal relapse of the mandible could also be attributed to the inaccuracies in the positioning of the condyles during the acquisition of one-year postoperative CBCT scans.

Although, the sequence of the performed osteotomies did not appear to affect post-operative relapse, this study has shown an impact of jaw translations and rotations on one-year skeletal relapse, with the magnitude of surgical displacement and skeletal relapse of the maxilla and mandible comparable to previous studies^26,27^.

This suggests that surgical jaw movements are an important contributor in skeletal relapse, and that a larger surgical movement and a CCW rotation of the bimaxillary complex increases the soft tissue and muscular tensions surrounding the jaws. This agrees with the systematic review by Joss & Vassalli (2009) who have shown an increased vertical relapse in patients with a low mandibular plane angle, and an increased horizontal relapse in patients with high mandibular plane angle^7^. Thus, this study coupled to the findings of previous research^15,19,28,29^ has indicated that pronounced skeletal relapse occurs when increased force is exerted on the jaw segments in the opposite direction of the desired movement.

An advantage of the present study is the utilisation of the newly developed and clinically validated OrthoGnathicAnalyser (OGA)^30^. The non-profit OGA software was developed at the 3D lab in Radboud University Medical Centre (Nijmegen, the Netherlands) by the authors. This analysis method was used to evaluate the patient’s postoperative skeletal relapse. This differs from majority of previous research^31,32^, in which linear and angular measurements on (2D) lateral cephalograms were used to assess the postoperative skeletal relapse. In contrast to all conventional 2D and 3D cephalometric analyses, the OGA eliminates the necessity of identifying anatomical landmarks multiple times^30^. By overcoming the landmark identification error, the OGA is an observer independent, semi-automatic tool, which is able to analyse the accuracy of the 3D planning and surgical outcome in an objective, reproducible and clinically relevant way. In a recently published systematic review, this tool was reported as currently the best method for assessing planning accuracy^33^. The drawback of the OGA was that it was software dependent and could only used with Maxilim® planning software. In the past year, the 3D lab has made progress in updating the OGA software. At this moment OGA is no longer software or platform dependent and can operate on any computer anywhere in the world.

A limitation of this study is the clinical study design. The ideal study design to evaluate the influence of sequencing bimaxillary osteotomies and the stability of 3D planning is a randomized controlled trial, having patients who are randomly assigned to the maxilla-first and mandible-first groups, while controlling all possible covariates. However, in clinical practice, this ideal study design may encounter grave ethical issues. Therefore, this retrospective cohort study has been set up. The clinical protocol and principles of 3D planning were identical in both groups.

With respect to our previous study^4^, which investigated the effects of sequencing bimaxillary osteotomies (maxilla-first versus mandible-first) on the achievability of the 3D virtually planned bimaxillary surgeries, it can be concluded that the sequence of surgery is more of clinical importance to the achievability of the 3D virtually planned repositioning of the jaws, rather than the stability of the achieved postoperative results. It is the surgeon’s choice to choose the most suitable sequence of bimaxillary osteotomies in each individual case. Taking the results of both studies into account, the maxilla-first approach remains to be a reliable and predictable surgical approach for the correction of bimaxillary anomalies. In certain circumstances, such as a planned CCW rotation of both jaws, the mandible-first sequence tends to result in more predictable displacement of the jaws. Overall, this study has shown that 3D virtual planning in combination with an optimised sequencing of osteotomies provides long-term predictable results in bimaxillary surgery.

## Patients and Methods

Patients who underwent bimaxillary osteotomies in the period from 2010 to 2014 at the Department of Oral and Maxillofacial Surgery in Radboud University Nijmegen Medical Centre were included in this study. The inclusion criteria were a non-syndromatic dysgnathia requiring bimaxillary osteotomies with or without genioplasty and the availability of a CBCT scan before and one year after surgery. All patients received preoperative orthodontic treatment to align their teeth and had a minimum of 24 teeth. The exclusion criteria were previous history of facial trauma with fractures of facial bones, or a history of orthognathic surgery, except a SARME (Surgically Assisted Rapid Maxillary Expansion) procedure.

This study was conducted in compliance with the World Medical Association Declaration of Helsinki on medical research ethics. The approval of the institutional review board (CMO Arnhem-Nijmegen, #181/2005) and informed consent were obtained for this study. All patient data were anonymized and de-identified prior to analysis.

### Data acquisition

CBCT scans were acquired four weeks prior to surgery and within one year after bimaxillary surgery using a standard CBCT scanning protocol (i-CAT, 3D Imaging System, Imaging Sciences International Inc, Hatfield, PA, USA) in “Extended Field” modus (FOV: 16 cm diameter/22 cm height; scan time: 2 × 20 s; voxel size: 0.4 mm). Patients were scanned while seated in natural head position. They were asked to swallow, to relax their lips and facial muscles and to keep their eyes open. The acquired CBCT data were exported in DICOM format and imported into Maxilim® software (Medicim NV, Mechelen, Belgium).

### Surgical planning

In Maxilim®, a 3D virtual augmented head model was rendered and positioned in a reference frame as described by Swennen *et al*.^34^. Subsequently, virtual osteotomies were performed to simulate the Le Fort I and BSSO osteotomies. The maxillary and mandibular segments were positioned into the desired positions in order to create a harmonious 3D soft tissue facial profile, as simulated in real-time by the Maxilim® software using the mass tensor model based soft tissue simulation^35^. If the facial profile required, an additional virtual chin osteotomy was simulated. Based on the 3D virtual planning, one intermediate and one final interocclusal splint were milled to transfer the virtual planning to the patient in the operating theatre.

Between 2010–2012 the clinical protocol for bimaxillary osteotomies was to start with the BSSO that was followed by the Le Fort 1 (mandible-first). After 2012 this protocol was changed and the Le Fort 1 was performed prior to the BSSO (maxilla-first).

### Surgical procedure

All bimaxillary osteotomies were performed or supervised by one experienced surgeon (MdK). After nasotracheal intubation, the mucobuccal fold of the maxilla and the mandibular ramus regions were infiltrated with local anaesthetic (Ultracain Ds-Forte). In cases of mandible-first procedure, a BSSO was performed according to the Hunsuck modification (Hunsuck, 1968)^36^. After the completion of the osteotomies using osteotomes, the distal segment of the mandible was placed in the planned position using the prefabricated interocclusal intermediate splint and stabilized with intermaxillary fixation (IMF). The proximal segments were gently pushed backward and upward to seat the condyles. The mandibular segments were fixed with two titanium miniplates (one on each side) and monocortical screws (Champy 2.0 mm, KLS Martin, Tuttlingen, Germany). Following the BSSO, a Le Fort I procedure was performed. After an incision in the gingivobuccal sulcus and elevation of mucoperiosteum and nasal mucosa, the osteotomies were made with a reciprocal saw at the Le Fort I level. The lateral nasal walls and nasal septum were osteotomized with a nasal osteotome. The piriform aperture and nasal spine were rounded. After mobilization of the maxilla, it was positioned in the planned position using a prefabricated final interocclusal splint. Fixation was performed with four 1.5 mm miniplates (KLS Martin, Tuttlingen, Germany) and 4 mm screws, one paranasal and one on the maxillary buttress on each side. Alar cinch suture and VY sutures were used accordingly. The mucosa was closed with a 3–0 Vicryl suture (Ethicon, Johnson and Johnson Medical, Norderstedt, Germany). In cases of maxillary first procedure, the Le Fort I osteotomy was carried out first, after which the BSSO was performed. The surgical protocol and method of fixation were identical as described in the mandible-first procedure.

### Postsurgical protocol

Depending on the stability of the occlusion, the final interocclusal splint was removed or left in place. Tight elastics were applied during the first postoperative week to keep a proper occlusion. After the first week, these elastics were replaced by guiding elastics, and were maintained for approximately two weeks. Postoperative orthodontic treatment occurred between three to four weeks after surgery.

### 3D analysis of 3D planned and 1-year postoperative positioning of jaws

The accuracy of the one-year postoperative surgical result was compared to the postoperative result and evaluated using the following steps.

Step 1: The 3D rendered pre- and postoperative 3D virtual head models were aligned by using voxel-based registration upon the anterior cranial base^37,38^.

Step 2: Virtual triangles were constructed on the maxilla and distal mandibular segment by using previously validated cephalometric landmarks^39^.

Step 3: The preoperative virtually osteotomized maxilla and distal mandibular segment were translated to the 3D planned position in Maxilim® by voxel-based registration. The landmarks placed on the preoperative maxilla and mandible, and thus the previously constructed triangles were translated along with the maxilla and mandible to the 3D planned position. The coordinates of the triangles were imported into the OGA^30^ to compute the 3D planned sagittal, vertical and transverse translations as well as rotations (pitch, roll and yaw) of the maxilla and distal mandibular segment.

**Figure 3:**
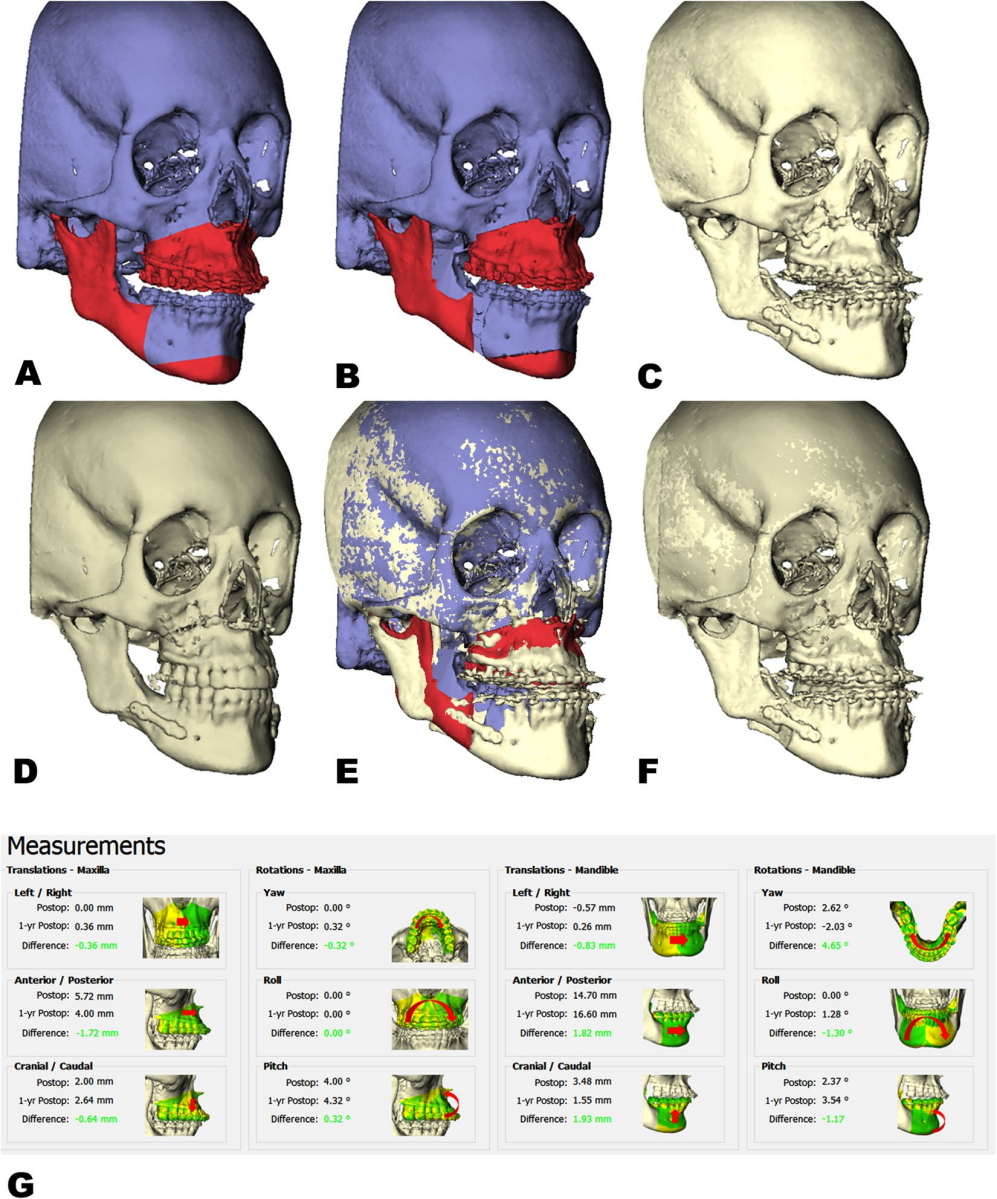
Example of a patient who underwent bimaxillary surgery. (**A**) Preoperative CBCT scan of the patient. (**B**) Virtual 3D planning of the bimaxillary complex in the planned position. (**C**) One-week postoperative CBCT after bimaxillary surgery. (**D**) One-year postoperative CBCT scan. (**E**) The one-week postoperative CBCT is superimposed on the planning using voxel-based registration of the head models on the anterior cranial base. (**F**) The one week postoperative CBCT is superimposed on one-year CBCT using voxel-based registration of the head models on the anterior cranial base. (**G**) Differences between the post surgical movement and one-year position of the maxilla, distal and proximal mandibular segments were calculated and displayed by the OrthoGnathicAnalyser.

Step 4: The maxilla and mandibular segments were again translated from the 3D planned position to the postoperative position through voxel-based registration, which resulted in a displacement of the virtual triangle. The coordinates of the landmarks (virtual triangle) in the postoperative position were imported into the OGA. The translational and rotational differences of the maxilla and distal mandibular segment between the actual postoperative surgical results and the one-year postoperative surgical results were calculated^30^ (Fig. 3).

### Statistical analysis

Statistical data analyses were performed with SPSS 23 for Windows (IBM Corp., Armonk, NY, USA). Mean relapse (difference) was calculated for both the mandible and maxilla in six different planes: translation – horizontal (anterior/posterior), lateral (left/right), vertical (up/down); rotation – pitch (CW/ CCW), roll (CW/CCW) and yaw (CW/CCW) (Figs 1 and 2.). All rotations were measured in degrees (°) and all translations in millimetres (mm). A one-way ANOVA and paired t-tests were used to assess the postoperative relapse between the directly postoperative and 1-year postoperative CBCT scans, based on the 5% level of significance (p ≤ 0.05). To evaluate the influence of the different directions on relapse, differences between the mean relapse of opposite directions (CW/CCW, anterior/posterior, left/right and up/down) were compared by using one-way ANOVA and were shown with a 5% level of significance. Univariate regression analyses were performed to identify the influence of different patient variables and operation variables on relapse. These results were shown as partial eta squared (partial η^2^), which is the proportion of variance accounted for by individual variables.

## Data Availability

The datasets generated during and/or analysed during the current study are available from the corresponding author on reasonable request.

## References

[1] Perez D, Ellis E (2011). Sequencing bimaxillary surgery: mandible first. Journal of oral and maxillofacial surgery: official journal of the American Association of Oral and Maxillofacial Surgeons.

[2] Perez D, Ellis E (2016). Implications of Sequencing in Simultaneous Maxillary and Mandibular Orthognathic Surgery. Atlas of the oral and maxillofacial surgery clinics of North America.

[3] Ritto FG, Ritto TG, Ribeiro DP, Medeiros PJ, de Moraes M (2014). Accuracy of maxillary positioning after standard and inverted orthognathic sequencing. Oral surgery, oral medicine, oral pathology and oral radiology.

[4] Liebregts J (2017). Achievability of 3D planned bimaxillary osteotomies: maxilla-first versus mandible-first surgery. Scientific reports.

[5] Turvey T (2011). Sequencing of two-jaw surgery: the case for operating on the maxilla first. Journal of oral and maxillofacial surgery: official journal of the American Association of Oral and Maxillofacial Surgeons.

[6] Stokbro K, Aagaard E, Torkov P, Bell RB, Thygesen T (2014). Virtual planning in orthognathic surgery. International journal of oral and maxillofacial surgery.

[7] Joss CU, Vassalli IM (2009). Stability after bilateral sagittal split osteotomy advancement surgery with rigid internal fixation: a systematic review. Journal of oral and maxillofacial surgery: official journal of the American Association of Oral and Maxillofacial Surgeons.

[8] Arnett GW, Milam SB, Gottesman L (1996). Progressive mandibular retrusion–idiopathic condylar resorption. Part I. Am J Orthod Dentofacial Orthop.

[9] Mobarak KA, Espeland L, Krogstad O, Lyberg T (2001). Mandibular advancement surgery in high-angle and low-angle class II patients: different long-term skeletal responses. Am J Orthod Dentofacial Orthop.

[10] Kim YJ (2014). Condylar positional changes up to 12 months after bimaxillary surgery for skeletal class III malocclusions. Journal of oral and maxillofacial surgery: official journal of the American Association of Oral and Maxillofacial Surgeons.

[11] Xi T, de Koning M, Berge S, Hoppenreijs T, Maal T (2015). The role of mandibular proximal segment rotations on skeletal relapse and condylar remodelling following bilateral sagittal split advancement osteotomies. J Craniomaxillofac Surg.

[12] Mucedero M, Coviello A, Baccetti T, Franchi L, Cozza P (2008). Stability factors after double-jaw surgery in Class III malocclusion. A systematic review. Angle Orthod.

[13] Jakobsone G, Stenvik A, Sandvik L, Espeland L (2011). Three-year follow-up of bimaxillary surgery to correct skeletal Class III malocclusion: stability and risk factors for relapse. Am J Orthod Dentofacial Orthop.

[14] Hoppenreijs TJ, Stoelinga PJ, Grace KL, Robben CM (1999). Long-term evaluation of patients with progressive condylar resorption following orthognathic surgery. International journal of oral and maxillofacial surgery.

[15] Borstlap WA, Stoelinga PJ, Hoppenreijs TJ, van’t Hof MA (2004). Stabilisation of sagittal split advancement osteotomies with miniplates: a prospective, multicentre study with two-year follow-up. Part III–condylar remodelling and resorption. International journal of oral and maxillofacial surgery.

[16] Eggensperger N, Smolka K, Luder J, Iizuka T (2006). Short- and long-term skeletal relapse after mandibular advancement surgery. International journal of oral and maxillofacial surgery.

[17] Han JJ, Yang HJ, Lee SJ, Hwang SJ (2014). Relapse after SSRO for mandibular setback movement in relation to the amount of mandibular setback and intraoperative clockwise rotation of the proximal segment. J Craniomaxillofac Surg.

[18] Proffit, W. R., Turvey, T. A. & Phillips, C. The hierarchy of stability and predictability in orthognathic surgery with rigid fixation: an update and extension. *Head & Face Medicine* (2007). 10.1186/1746-160X-3-21 PMC1876453 17470277

[19] Hernandez-Alfaro F (2017). Three-Dimensional Analysis of Long-Term Stability After Bilateral Sagittal Split Ramus Osteotomy Fixed With a Single Miniplate With 4 Monocortical Screws and 1 Bicortical Screw: A Retrospective 2-Center Study. Journal of oral and maxillofacial surgery: official journal of the American Association of Oral and Maxillofacial Surgeons.

[20] Chen S (2013). Short- and long-term changes of condylar position after bilateral sagittal split ramus osteotomy for mandibular advancement in combination with Le Fort I osteotomy evaluated by cone-beam computed tomography. Journal of oral and maxillofacial surgery: official journal of the American Association of Oral and Maxillofacial Surgeons.

[21] Kim YI, Cho BH, Jung YH, Son WS, Park SB (2011). Cone-beam computerized tomography evaluation of condylar changes and stability following two-jaw surgery: Le Fort I osteotomy and mandibular setback surgery with rigid fixation. Oral Surg Oral Med Oral Pathol Oral Radiol Endod.

[22] Ayoub AF, Trotman CA, Stirrups DR, Wilmot JJ (1997). Stability of bimaxillary osteotomy following surgical correction of class II skeletal deformities: a two-centre study. The British journal of oral & maxillofacial surgery.

[23] Xi T (2017). Three-dimensional analysis of condylar remodeling and skeletal relapse following bimaxillary surgery: A 2-year follow-up study. Journal of cranio-maxillo-facial surgery: official publication of the European Association for Cranio-Maxillo-Facial Surgery.

[24] Ayoub AF, Stirrups DR, Moos KF (1993). The stability of bimaxillary osteotomy after correction of skeletal Class II malocclusion. The International journal of adult orthodontics and orthognathic surgery.

[25] Moen K, Wisth PJ, Skaale S, Boe OE, Tornes K (2011). Dental or skeletal relapse after sagittal split osteotomy advancement surgery? Long-term follow-up. Journal of oral and maxillofacial surgery: official journal of the American Association of Oral and Maxillofacial Surgeons.

[26] Arpornmaeklong P, Shand JM, Heggie AA (2004). Skeletal stability following maxillary impaction and mandibular advancement. International journal of oral and maxillofacial surgery.

[27] Schwartz K, Rodrigo-Domingo M, Jensen T (2016). Skeletal Stability after Large Mandibular Advancement (>10 mm) with Bilateral Sagittal Split Osteotomy and Skeletal Elastic Intermaxillary Fixation. Journal of oral & maxillofacial research.

[28] Joss CU, Thuer UW (2008). Stability of the hard and soft tissue profile after mandibular advancement in sagittal split osteotomies: a longitudinal and long-term follow-up study. Eur J Orthod.

[29] Van Sickels JE (2000). Technical factors accounting for stability of a bilateral sagittal split osteotomy advancement: wire osteosynthesis versus rigid fixation. Oral Surg Oral Med Oral Pathol Oral Radiol Endod.

[30] Baan F (2016). A New 3D Tool for Assessing the Accuracy of Bimaxillary Surgery: The OrthoGnathicAnalyser. PloS one.

[31] Park KH, Sandor GK, Kim YD (2016). Skeletal stability of surgery-first bimaxillary orthognathic surgery for skeletal class III malocclusion, using standardized criteria. International journal of oral and maxillofacial surgery.

[32] Kor HS, Yang HJ, Hwang SJ (2014). Relapse of skeletal class III with anterior open bite after bimaxillary orthognathic surgery depending on maxillary posterior impaction and mandibular counterclockwise rotation. Journal of cranio-maxillo-facial surgery: official publication of the European Association for Cranio-Maxillo-Facial Surgery.

[33] Gaber, R. M. *et al*. A Systematic Review to Uncover a Universal Protocol for Accuracy Assessment of 3-Dimensional Virtually Planned Orthognathic Surgery. *Journal of oral and maxillofacial surgery: official journal of the American Association of Oral and Maxillofacial Surgeons*, 10.1016/j.joms.2017.05.025 (2017). 10.1016/j.joms.2017.05.025 28646644

[34] Swennen GR (2009). A cone-beam computed tomography triple scan procedure to obtain a three-dimensional augmented virtual skull model appropriate for orthognathic surgery planning. The Journal of craniofacial surgery.

[35] Mollemans W, Schutyser F, Nadjmi N, Maes F, Suetens P (2007). Predicting soft tissue deformations for a maxillofacial surgery planning system: from computational strategies to a complete clinical validation. Med Image Anal.

[36] Hunsuck EE (1968). A modified intraoral sagittal splitting technic for correction of mandibular prognathism. Journal of oral surgery (American Dental Association: 1965).

[37] Maes F, Collignon A, Vandermeulen D, Marchal G, Suetens P (1997). Multimodality image registration by maximization of mutual information. IEEE transactions on medical imaging.

[38] Nada RM (2011). Accuracy and reproducibility of voxel based superimposition of cone beam computed tomography models on the anterior cranial base and the zygomatic arches. PloS one.

[39] Swennen GR, Schutyser F, Barth EL, De Groeve P, De Mey A (2006). A new method of 3-D cephalometry Part I: the anatomic Cartesian 3-D reference system. The Journal of craniofacial surgery.

