# Author Correction: One-year postoperative skeletal stability of 3D planned bimaxillary osteotomies: maxilla-first versus mandible-first surgery

**DOI:** 10.1038/s41598-020-76584-3

**Published:** 2020-11-04

**Authors:** Jeroen Liebregts, Frank Baan, Pieter van Lierop, Martien de Koning, Stefaan Bergé, Thomas Maal, Tong Xi

**Affiliations:** 1 Department of Oral and Maxillofacial Surgery, Radboud University Nijmegen Medical Centre, Geert Grooteplein 10, 6525 GA Nijmegen, The Netherlands; 2 Department of Orthodontics and Craniofacial Biology, Radboud University Nijmegen Medical Centre, Philips van Leydenlaan 25, 6525 EX Nijmegen, The Netherlands; 3 Radboudumc 3D Lab, Radboud University Medical Center, Nijmegen, The Netherlands

Correction to: *Scientific Reports* 10.1038/s41598-019-39250-x, published online 28 February 2019

This Article contains numerical errors in Table 2, whereby some p-values are incorrectly published as the mean and standard deviation under the heading ‘Relapse (1wk-1 yr) Mean ± SD’. The correct Table 2 appears below as Table 1.

**Table 1:** Translations and rotations of the maxilla and mandible after 1 week, 1 year and the postoperative relapse. Translations are given in millimetres, rotations are given in degrees.

		Maxilla					Mandible				
		n	CBCT 1 wk Mean ± SD	CBCT 1 yr Mean ± SD	Relapse (1wk-1 yr) Mean ± SD	*p* value	n	CBCT 1 wk Mean ± SD	CBCT 1 yr Mean ± SD	Relapse (1wk-1 yr) Mean ± SD	*p* value
**Translation**											
X	Left	51	1.4 ± 1.1	1.3 ± 1.2	0.2 ± 0.9	0.20	62	1.6 ± 1.5	1.1 ± 1.7	0.5 ± 1.3	**0.00**
	Right	55	1.2 ± 1.2	1.1 ± 1.2	0.2 ± 0.8	0.11	44	1.9 ± 1.8	1.3 ± 2.0	0.7 ± 2.0	0.04
Y	Anterior	97	3.3 ± 2.1	3.1 ± 2.1	0.2 ± 1.3	0.19	92	8.1 ± 3.8	7.6 ± 3.2	0.5 ± 2.3	0.05
	Posterior	9	0.7 ± 0.5	0.5 ± 1.2	0.2 ± 1.2	0.56	14	3.1 ± 1.4	1.3 ± 1.9	1.8 ± 1.2	**0.00**
Z	Caudal	57	2.8 ± 2.0	2.1 ± 1.9	0.7 ± 1.4	**0.00**	52	3.2 ± 1.9	1.8 ± 2.3	1.4 ± 2.0	**0.00**
	Cranial	48	3.0 ± 2.3	2.2 ± 2.3	0.7 ± 1.1	**0.00**	54	3.0 ± 2.4	2.2 ± 2.8	0.8 ± 2.0	**0.00**
**Rotation**											
Pitch	CCW	58	3.0 ± 2.7	2.0 ± 2.7	1.0 ± 1.3	**0.00**	70	3.9 ± 3.1	1.7 ± 2.9	2.3 ± 2.6	**0.00**
	CW	48	3.5 ± 2.5	2.6 ± 2.5	0.9 ± 1.6	**0.00**	36	4.0 ± 3.1	3.2 ± 3.1	0.8 ± 1.9	**0.02**
Roll	CCW	52	1.6 ± 1.5	1.1 ± 1.5	0.4 ± 0.7	**0.00**	46	1.5 ± 1.5	0.9 ± 1.4	0.6 ± 1.1	**0.00**
	CW	54	1.1 ± 0.8	0.8 ± 1.0	0.3 ± 0.8	**0.01**	59	1.2 ± 1.1	0.5 ± 0.9	0.8 ± 1.1	**0.00**
Yaw	CCW	52	1.3 ± 1.0	1.0 ± 1.1	0.3 ± 0.6	**0.00**	57	1.3 ± 1.0	1.1 ± 1.4	0.2 ± 1.3	0.20
	CW	53	1.1 ± 1.1	0.9 ± 1.2	0.2 ± 0.8	**0.03**	49	1.6 ± 1.5	1.3 ± 1.6	0.4 ± 1.1	**0.03**

CBCT: Cone-Beam Computed Tomography, SD: Standard Deviation, 1wk: one week, 1 yr: one year, CW: Clockwise, CCW: Counterclockwise.

